# Causal effect of waist-to-hip ratio on non-alcoholic fatty liver disease: a mendelian randomization study

**DOI:** 10.3389/fgene.2024.1414835

**Published:** 2024-11-19

**Authors:** Shihao Wu, Yuhong He, Jiaxing Li, Sijie Wang

**Affiliations:** ^1^ Guangdong Medical University, Zhanjiang, China; ^2^ Hepatobiliary Surgery Laboratory, Affiliated Hospital of Guangdong Medical University, Zhanjiang, China; ^3^ Clinical Research and Experimental Center, Affiliated Hospital of Guangdong Medical University, Zhanjiang, China

**Keywords:** NAFLD, waist-to-hip ratio, mendelian randomization, genetic variants, causal relationship

## Abstract

**Objective:**

This study aimed to explore the potential causal association between waist-to-hip ratio (WHR) and the risk of non-alcoholic fatty liver disease (NAFLD) via the Mendelian randomization (MR) approach.

**Methods:**

Genetic variation data pertaining to WHR served as instrumental variables, while genome-wide association study data for NAFLD constituted the outcome event. Primarily, the random-effects inverse-variance weighted (IVW) method was utilized, supplemented by MR Egger, weighted median, simple mode, and weighted mode analyses. Sensitivity analysis entailed the “leave-one-out” approach, with the IVW results forming the foundational basis for this study.

**Results:**

This analysis included a total of 28 valid single nucleotide polymorphisms (SNPs). IVW analysis indicated an increased risk of NAFLD associated with WHR (OR = 1.61; 95% CI: 1.08–2.41; P = 0.02). Furthermore, MR-Egger regression analysis revealed the absence of horizontal pleiotropy among the included SNPs, albeit with some sample heterogeneity. Lastly, the “leave-one-out” sensitivity analysis demonstrated that no individual SNP significantly influenced the estimated causal association.

**Conclusion:**

This study furnishes indicative evidence of a causal link between waist-to-hip ratio and the risk of NAFLD occurrence.

## 1 Introduction

Non-alcoholic fatty liver disease (NAFLD), a prevalent chronic liver disease globally, imposes a considerable health burden on diverse populations. Recent statistics indicate that its global prevalence among adults has reached 25% (Bebawi et al., 2023) NAFLD encompasses a spectrum of liver conditions, including nonalcoholic simple fatty liver, nonalcoholic steatohepatitis, and related cirrhosis (Friedman et al., 2018), this condition is identified by the accumulation of fat within liver cells, specifically in the form of large droplets, without a history of alcohol misuse. While fatty liver and ongoing inflammation are recognized as the primary drivers of NAFLD, the underlying molecular biology remains intricate and is not completely clear (Tilg et al., 2021). If left untreated, these conditions may progress to cirrhosis and liver cancer, resulting in more severe health complications (Schuster et al., 2018). Numerous studies have conclusively linked NAFLD with various lifestyle factors, including alcohol abuse (Wongtrakul et al., 2021), smoking (Akhavan Rezayat et al., 2018) and coffee consumption (Chen et al., 2019). Furthermore, NAFLD frequently coexists with metabolic disorders such as obesity (Quek et al., 2023), type 2 diabetes (Younossi et al., 2019) and hyperlipidemia. Given this complex interplay between NAFLD and lifestyle habits, rational dietary and exercise interventions aimed at lifestyle optimization are essential and critical components of NAFLD treatment. Moreover, adjunctive pharmacotherapy can regulate glucose and lipid metabolism, thereby reducing liver inflammation and fibrosis and advancing the treatment of NAFLD (Rong et al., 2022). However, current NAFLD treatment predominantly relies on dietary and exercise interventions, with a notable dearth of specific and effective pharmacological options. Therefore, our objective is to identify risk factors associated with NAFLD, elucidate causal relationships between these factors and disease progression, and initiate appropriate interventions and preventive measures.

This approach promises to enhance NAFLD management and patient outcomes, ultimately mitigating the burden of this chronic liver disease on both individuals and society. Numerous observational studies have solidly established a robust correlation between obesity and NAFLD. In recent years, waist-hip ratio (WHR) has gained increasing popularity as a metric for assessing obesity. Compared to body mass index (BMI), WHR offers a more precise indication of adverse abdominal fat accumulation and is simpler to calculate (Huxley et al., 2010). Recent studies have demonstrated a close association between waist-to-hip ratio and the risk of various chronic diseases, including cardiovascular disease, lower back pain, and myocardial infarction. However, a significant research gap exists in directly linking WHR to NAFLD (Huxley et al., 2010; Cao et al., 2018; You et al., 2022). Waist-to-hip ratio, the ratio of waist to hip circumference, is a vital indicator for assessing central obesity. Epidemiological surveys indicate that the predominant body fat distribution pattern in China is central obesity. Importantly, NAFLD has been escalating in China, correlating with the prevalence of central obesity, thus becoming the country’s most common chronic liver disease (Zhou et al., 2020). Our goal is to elucidate the causal relationship between WHR and NAFLD risk employing a reliable methodology.

Mendelian randomization studies present a robust and innovative method for inferring causality. Leveraging genetic variations as instrumental variables, these studies facilitate exploration of the causal link between exposures and outcomes. The relative stability of genetic effects, less susceptible to environmental factors, allows Mendelian randomization studies to effectively circumvent confounding factors and reverse causality issues commonly encountered in traditional observational epidemiology (Sekula et al., 2016). Owing to these advantages, this approach has found widespread application in various studies (Bowden and Holmes, 2019). In this study, we utilized Mendelian randomization to analyze the causal relationship between waist-hip ratio and NAFLD risk. Our objective is to provide a valuable reference for the early clinical identification of NAFLD, contributing to the prevention and management of this chronic liver disease.

## 2 Materials and methods

### 2.1 Study design

**FIGURE 1:**
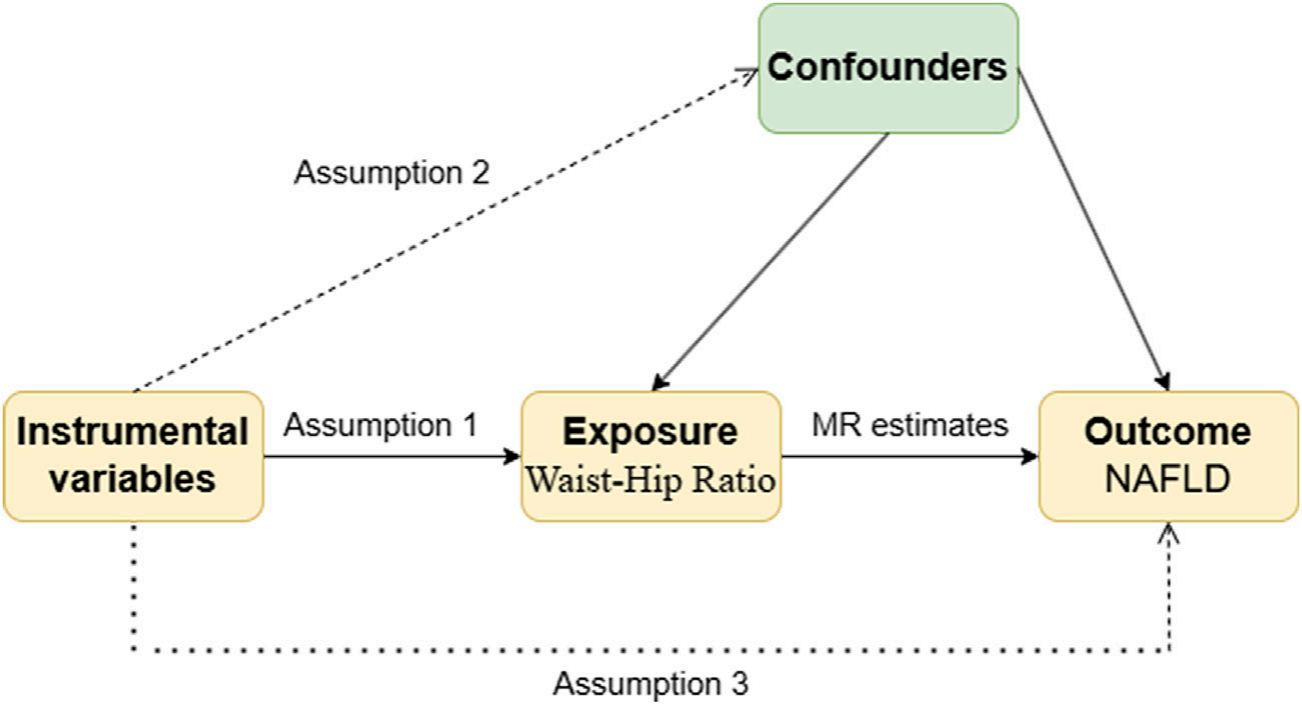
Schematic diagram for MR analysis.

The present study employed a two-sample Mendelian randomization approach, utilizing publicly available summary-level genome-wide association study (GWAS) data to investigate the causal association between waist-hip ratio (WHR) and NAFLD risk. The implementation of this study adhered to the three fundamental assumptions of Mendelian randomization, the analysis flow is shown in Figure 1: (1) Association assumption: the instrumental variable (genetic variant, IV) should be strongly associated with the exposure factor, indicating a robust association between SNPs related to WHR and WHR itself; (2) Independence assumption: the instrumental variable should be independent of confounding factors; (3) Exclusivity assumption: the instrumental variable should only act on the outcome through the exposure, implying no pleiotropy, that is, the SNP serving as the instrumental variable can only influence NAFLD risk through WHR.

### 2.2 Data sources

All genetic data used in this study were retrieved from the IEU open GWAS database (https://gwas.mrcieu.ac.uk/). The GWAS data for the exposure factor of waist-hip ratio (id: ieu-a-73) were published in 2015 (Shungin et al., 2015), encompassing 2,124,448 European individuals and 2,560,782 genetic variants (SNPs). The GWAS data for NAFLD as the outcome event (id: ebi-a-GCST90091033) were published in 2021 (Ghodsian et al., 2021), including 778,614 European individuals (8,434 cases and 770,180 controls) with a total of 6,784,388 SNPs.

### 2.3 Selection of instrumental variables

After obtaining the GWAS summary data for waist-hip ratio and NAFLD, we filtered for strongly associated single nucleotide polymorphisms (SNPs) with both traits as instrumental variables (IV), using a significance threshold of P < 5 × 10^−8^. SNPs strongly associated with waist-hip ratio were prioritized as IVs. To ensure independence among the IVs for the exposure, linkage disequilibrium (LD) was set at r^2^ < 0.001, with a genetic distance of 10,000 kb. Effect allele frequencies were harmonized across the exposure and outcome datasets, excluding palindromic SNPs with intermediate allele frequencies. Additionally, the F statistic was calculated to assess the potential for weak instrument bias among the selected IVs; an F > 10 indicated the absence of weak instrument bias, further validating the association assumption.

### 2.4 Statistical analysis

To investigate the causal association between waist-hip ratio and NAFLD, we conducted Mendelian randomization (MR) analysis using five different approaches: inverse-variance weighted (IVW) as the primary method, and MR Egger, weighted median, simple mode, and weighted mode as supplementary methods. The IVW results served as the primary basis for this study. While each genetic variant satisfied the IVs assumptions, the IVW method employed a meta-analysis approach, incorporating Wald ratio estimates of causality derived from various SNPs and providing a robust assessment of the causal relationship between the exposure and outcome (Lee, 2019). In contrast to IVW, the MR-Egger method accounted for the presence of an intercept term, did not rely on non-zero average pleiotropy, but sacrificed statistical power (Zheng et al., 2021). The weighted median could provide a consistent estimate if at least 50% of the instrumental variables were valid in the analysis (Yavorska and Burgess, 2017).

We utilized Cochran’s Q statistic (for MR-ivw) and Rucker’s Q statistic (for MR Egger) to detect heterogeneity in the MR analysis, with p > 0.05 indicating the absence of heterogeneity (Hemani et al., 2018). Horizontal pleiotropy was tested using the global test from MR-PRESSO analysis, with p > 0.05 indicating the absence of horizontal pleiotropy. Our MR analysis employed the outlier test from MR-PRESSO analysis to detect potential outliers (Meng et al., 2022). Importantly, any outliers identified by the MR-PRESSO outlier test were excluded, and the causal estimates were reassessed. Additionally, a leave-one-out analysis was conducted to assess the potential influence of individual SNPs on the causal effect.

## 3 Results

### 3.1 Causal association between waist-hip ratio and NAFLD

**FIGURE 2:**
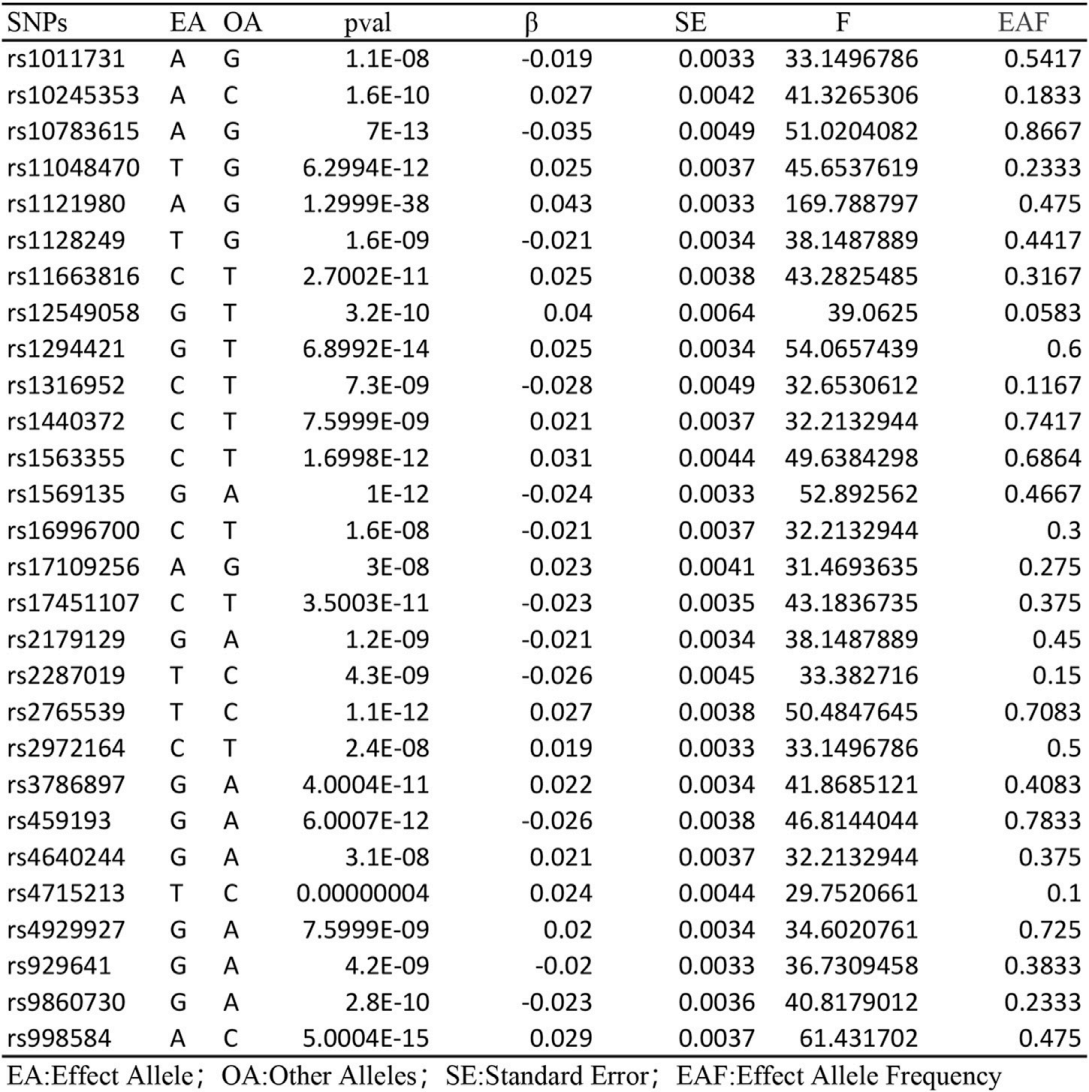
Instrumental variable characterization of waist-to-hip ratio.

**FIGURE 3:**
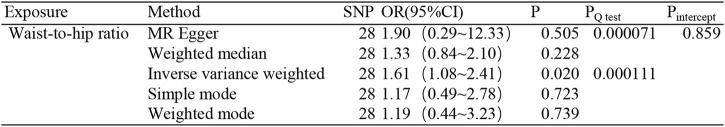
Results of the five MR analysis methods for investigating the causal association between waist-hip ratio and NAFLD.

**FIGURE 4:**
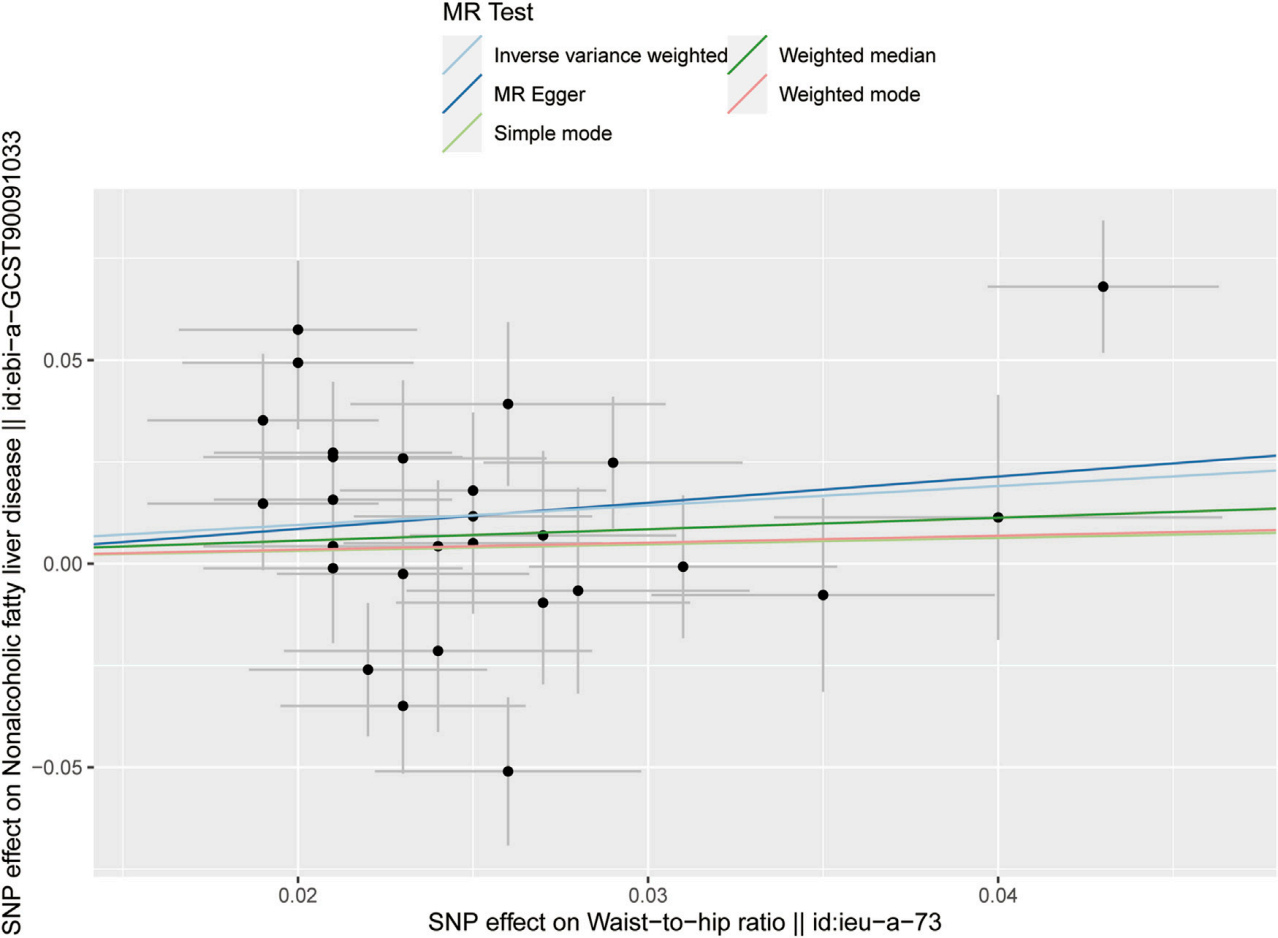
Scatter plot showing the results of five MR analysis methods for investigating the causal association between waist-hip ratio and NAFLD.

**FIGURE 5:**
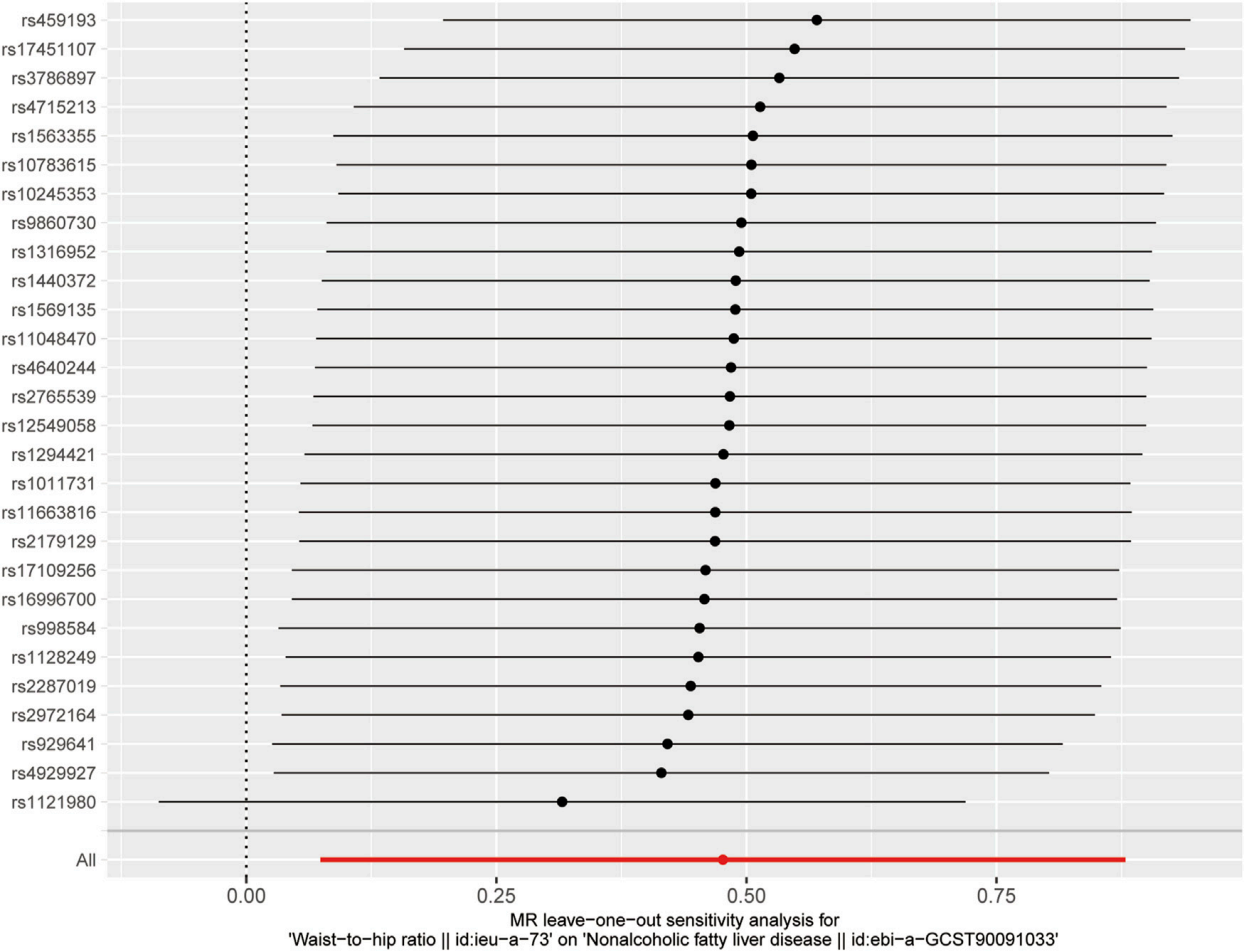
Results of the “leave-one-out” analysis for investigating the causal association between waist-hip ratio and NAFLD.

**FIGURE 6:**
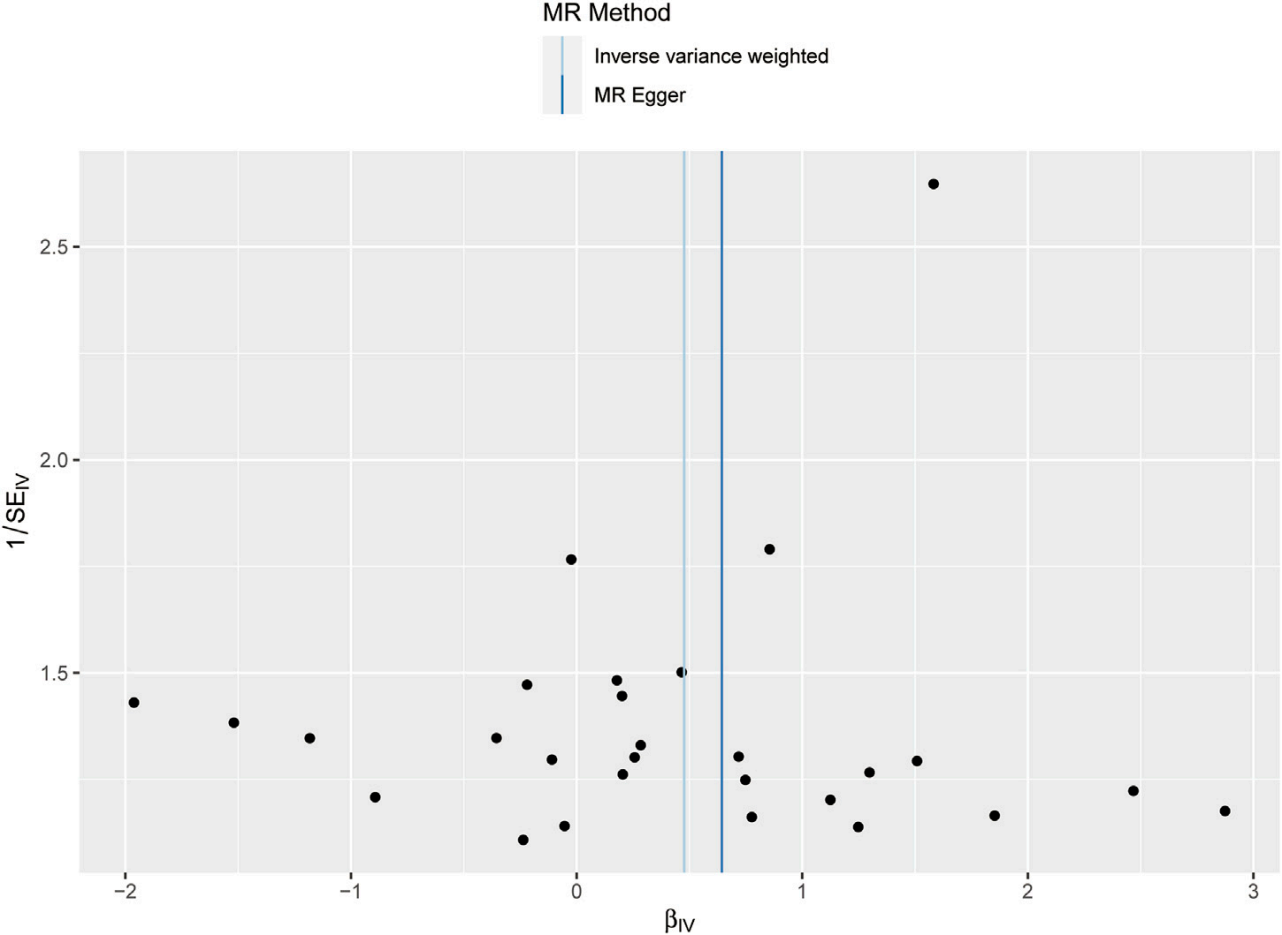
Funnel plot of the MR effect of waist-hip ratio on NAFLD.

Following the removal of LD through the screening of SNPs associated with exposure, 28 SNPs were identified as being associated with waist-to-hip ratio. The effect allele frequencies were employed to synchronize the exposure and outcome datasets for these SNPs, and palindromic SNPs with intermediate allele frequencies were excluded. The calculated F-statistics were all greater than 10 (Figure 2), indicating that there is no weak instrumental variable bias for these SNPs and that they can therefore be used as IVs. With WHR as the exposure and NAFLD as the outcome, results of the MR analysis are summarized in Figure 3. IVW analysis demonstrated a positive association between a higher waist-hip ratio and an elevated risk of NAFLD. Specifically, an increase in waist-hip ratio by one standard deviation correlated with a 61% increase in the risk of NAFLD (OR = 1.61; 95%CI: 1.08–2.41; P = 0.02). The MR-Egger analysis (Figure 4) revealed that the intercept term did not differ significantly from zero (P > 0.05), indicating an absence of horizontal pleiotropy. Cochran’s Q and Rucker’s Q tests, however, indicated statistically significant heterogeneity among the SNPs (P < 0.05), potentially attributable to variations in analysis platforms, experimental designs, populations, and analysts. The ‘leave-one-out’ sensitivity analysis (Figure 5) demonstrated that the exclusion of any individual SNP related to waist-hip ratio did not significantly change the IVW results compared to the overall analysis, suggesting that no single SNP exerted a disproportionate influence on the causal estimate. The funnel plot (Figure 6) exhibited a symmetric distribution of points, signifying no notable disparities among the included SNPs. Overall, these findings imply a causal association between waist-hip ratio and NAFLD risk, characterized by significant reliability and robustness.

## 4 Discussion

Non-alcoholic fatty liver disease (NAFLD) is increasingly recognized as a major health issue, with obesity identified as a primary risk factor. Our study introduces a novel genetic perspective by establishing, through Mendelian randomization and a publicly available GWAS database, a causal link between WHR and NAFLD risk. This discovery not only offers a clinically accessible method for risk assessment but also sets the stage for innovative preventative and therapeutic approaches.

Traditional epidemiological research has highlighted obesity’s strong association with NAFLD (Quek et al., 2023), yet clinical metrics have predominantly focused on body mass index (BMI), which fails to capture the nuances of fat distribution. We demonstrate that WHR—a more precise measure of adiposity—better predicts NAFLD risk, particularly abdominal fat accumulation, which is a significant predictor of metabolic diseases such as cardiovascular disease and diabetes. The simplicity of WHR measurement facilitates its clinical adoption, enhancing early identification of high-risk NAFLD populations and enabling timely interventions.

In Asian populations, including China, where obesity often presents as central or centripetal obesity, WHR emerges as a crucial health indicator. Our findings underscore the imperative to incorporate WHR into clinical assessments, thereby enabling clinicians to more accurately gauge patient health risks and devise targeted interventions.

The present study boasts several advantages: ① The employment of Mendelian randomization, an innovative statistical approach, showcases distinct strengths in epidemiological research, especially in delineating causal relationships between genotypes and phenotypes while circumventing potential confounding factors and reverse causality typical in traditional observational studies. ② Furthermore, we conducted a thorough evaluation of the causal association between waist-hip ratio and NAFLD risk using five distinct methodologies and utilized the “leave-one-out” approach to bolster the robustness and reliability of our findings. Despite these advantages, this study shares inevitable limitations with earlier Mendelian randomization studies (Huang et al., 2021; Levin and Burgess, 2024): ① The study population was confined to Europeans, which restricts the generalizability of the results (Yuzbashian et al., 2023) The research should enlarge the sample size to encompass diverse populations. ② Secondly, our use of aggregated data introduces limitations in accessing detailed patient characteristics (such as age, gender), lifestyle factors (such as physical activity), and disease status (such as severity), potentially impeding more detailed analyses (Burgess et al., 2013). ③While Mendelian randomization can surmount biases present in traditional epidemiological studies, Cochran’s Q test in this study indicated heterogeneity among SNPs, potentially arising from differences in analysis platforms, experimental procedures, populations, or analysts. Nevertheless, the SNPs included in this study constituted strong instrumental variables devoid of horizontal pleiotropy, ensuring the clinical relevance of the results.

In order to standardize and augment clinical focus on waist-hip ratio, additional observational studies are required to validate our findings. Future research should additionally consider NAFLD as an exposure and waist-hip ratio as an outcome, to further elucidate this relationship. However, the already established causal link between waist-hip ratio and NAFLD risk is of significant importance, underscoring the necessity of considering individual waist-hip ratio changes, in conjunction with traditional dietary and exercise factors, in the prevention and management of NAFLD.

## Data Availability

The datasets presented in this study can be found in online repositories. The names of the repository/repositories and accession number(s) can be found in the article/supplementary material.
